# Addressing HIV/AIDS in South African Classrooms

**DOI:** 10.1371/journal.pbio.1001390

**Published:** 2012-09-18

**Authors:** Lindsey Rabinowitz

**Affiliations:** eMPathy Trust southern Africa, Richmond, KwaZulu Natal, South Africa

## Abstract

In its commitment to HIV/AIDS prevention, Empathy Trust southern Africa develops teaching materials for schools such as the transmission game.

South Africa continues to have more people living with HIV and AIDS than any other country in the world. Raising awareness about how the disease is transmitted, and educating the public about prevention in particular, remain a major priority for several organisations. Despite these campaigns, the public's understanding of how to prevent sexual transmission of HIV has actually decreased in some areas of the country [1]. eMPathy Trust southern Africa works to advance public understanding of the disease by developing appropriate teaching materials and learner-centred teaching methods for school students between the ages of 12 and 14 years. The project was initiated by immunologist and native South African Professor Siamon Gordon, University of Oxford. Following a needs assessment, scientist and children's science writer Fran Balkwill, a professor of cancer and inflammation at Queen Mary University of London and director of Centre of the Cell, illustrator Mic Rolph, and publisher John Inglis of Cold Spring Harbour Press worked together to produce a children's book entitled *Staying Alive Fighting HIV/AIDS* in 2005. The book presented the biological facts about HIV/AIDS in an attempt to help counter the myths surrounding the virus and disease.

## Finding the Balance

*Staying Alive Fighting HIV/AIDS* was extensively field tested with relevant stakeholders. Based on the feedback and recommendations received, the book was rewritten and re-titled *You, Me & HIV*. The revised version maintains the scientific approach of the first book. It is based on the assumption that students will both deepen their scientific knowledge and be in a better position to make effective life choices if they understand the science behind the viral infection. The second version also emphasises the social and personal skills that are required to help students cope with the presence of HIV/AIDS in their lives [2]. At the request of teachers, an educator's guide and two posters were produced to accompany the textbook. The guide contains a wide selection of activities, using participatory teaching methods that encourage students to engage imaginatively with the content. It is intended for use by natural science, life orientation, and arts and culture teachers. The Trust has undertaken two sets of implementation evaluations of the guide, which were conducted by independent researchers. The evaluation methods were quantitative, such as written questionnaires that were completed by students and researchers, and qualitative, including workshop observation notes, focus groups, and semi-structured interviews with teachers and students. The recommendations from each helped revise and improve the subsequent training workshops.

eMPathy Trust is currently involved in running training programmes for teachers in-service or pre-service, in addition to developing materials and training for student peer educators.

## The Transmission Game: An Example of Our Approach in Practice

The transmission game is played as follows. Students are invited to a “party” where each participant is offered a glass of water and given an instruction on a piece of paper. The participants are warned not to drink from the glasses as the water may contain dangerous chemicals. One of the many glasses of water contains caustic soda. This represents the virus. Each student receives one of three possible instructions. Instruction one states that the participants may exchange fluids with as many people as possible. Instruction two states that the participant must find a partner and only exchange fluids with that person. The final instruction states that the participant should always say “no” to any request to exchange liquid. The students are given about five minutes to enact the instructions. The teacher then asks them to stop exchanging fluids and asks: “What do you think you have been doing?” The students will invariably answer that they have been engaging in unprotected sex. The teacher then invites the students to come forward for an HIV test. A drop of dye (phenolphthalein) is put into each glass. If the water turns pink, it indicates a positive HIV test result. Clear water indicates a negative result. The teacher reminds the students that only one glass did not contain pure water at the beginning of the activity.

The teacher follows the role play by leading a class discussion based on the following questions: How many of you tested positive? What were your instructions? How many of you tested negative? What were your instructions? How many people tested positive even though their instructions told them to share with only one partner or to refuse to exchange liquids? What is the rate of transmission in this group and why? What does this tell us about HIV transmission? The teacher elicits the different scenarios and behaviours that make a person susceptible to contracting HIV. The activity shows, for example, that one may be infected by an unfaithful partner even if one is faithful oneself. Although someone may refuse to have sex initially, they are often coerced into having it and may become positive as a result. The activity demonstrates that having multiple partners without using a condom increases the risk of contracting the virus.

The teacher then demonstrates the role of antiretroviral (ARV) treatment by dropping some vinegar (acetic acid) into a positive test result. The pink liquid will become clear. This represents an undetectable viral load and illustrates the efficacy of treatment. It could be argued that this part of the activity might deflect attention from the activity's chief message that HIV is easy to acquire but cannot be cured. In a climate of despair and stigma, it is important, however, to give students a sense of hope by emphasising the availability and effectiveness of treatment.

### Lessons Learned

Whilst this activity does not directly deal with the biology of HIV, it uses a scientific process as a metaphor and a method to convey information about a number of important principles related to the transmission of HIV, treatment of HIV/AIDS, and its prevention. It graphically illustrates the fact that the risk of HIV transmission is highest in the early phases of the disease when high viral loads are present. It also demonstrates that stable, monogamous relationships can prevent the spread of HIV, especially if both parties are regularly tested for HIV. Lastly, it demonstrates that the risk of contracting HIV exists for everyone who has sexual intercourse without a condom, that one cannot tell who has HIV, and that only tests can indicate a person's status. This activity should ideally be taught by a natural science teacher in collaboration with a life orientation teacher so that both the biology and the social content are given equal weight and attention. It is recommended that teachers adapt the activity to the particular context of their students.

### Anecdotal Evidence

The transmission game is played at training workshops followed by a written and verbal feedback process. In general, most teachers are eager to use the activity in their classroom and are confident about how to teach it. It is not uncommon for several teachers in a workshop to say that the activity has persuaded them to go for an HIV test. A group of teachers reported at a follow up meeting that 20 of their students volunteered to go for an HIV test after participating in the transmission game. Dr. Heinrich Heinrichs, a science teacher and education consultant who developed the activity while working at the Science Education Centre in Soweto between 1999 and 2002, observes that there was always a similar reaction when he revealed that one of the participants (a student, teacher, or even a top education management official) was “infected” and that they were obliged to come forward for a test:

Their faces always displayed dismay, always. There was never a time when someone did not take this seriously. My point about this activity is that it always gets people focused on the issue and nobody leaves without being touched. This activity is remembered after many years. The aim is to get students discussing all aspects of the activity and to let them make sense of it. This forms a constructivist approach: The teacher gives food for thought, and the student makes sense of it! (Heinrichs, 2012)

This sort of anecdotal evidence suggests that the activity is valuable and effective.

**Figure 1:**
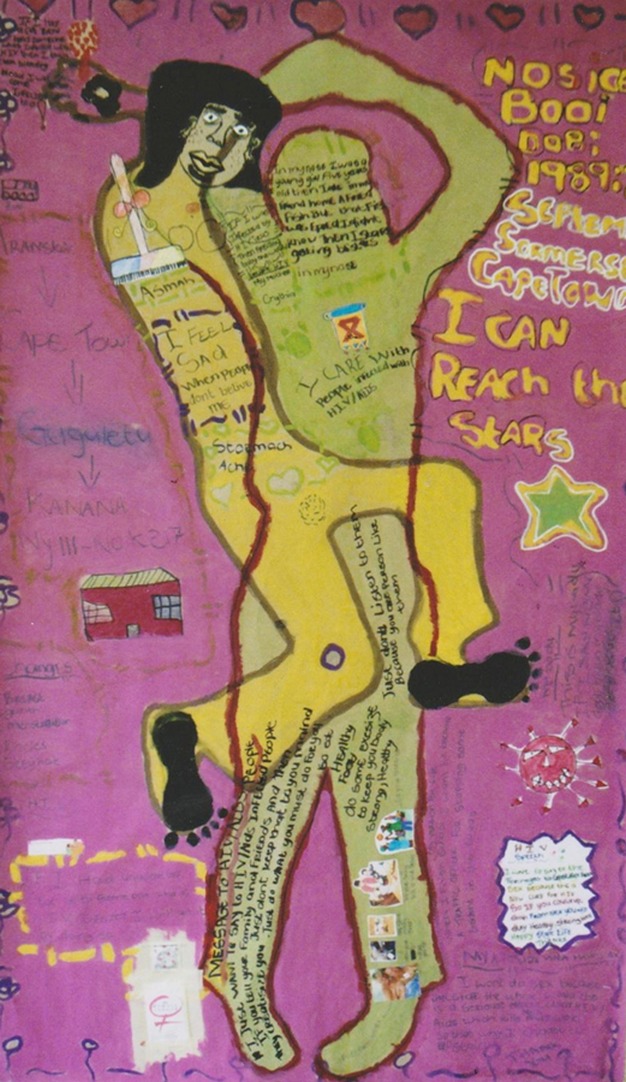
Body mapping. Body mapping is designed to help students negotiate and assert their personal boundaries in relation to their feelings and choices regarding their sexuality. (Photographer Kali van der Merwe.)

The activity is memorable as a result of the powerful nature of its central theme: finding out that one is either HIV positive or HIV negative. Though the transmission game is just one of several science-based activities in the training programme, it offers multiple entry points to discuss the complex questions surrounding HIV/AIDS prevention. Its effectiveness is greatly increased by its relationship to the activities that precede and follow it. For example, “Making the Immune System” teaches students about the biology of HIV by using clay or play dough and drama to describe microorganisms, their effect on the body, how the immune system works to protect the body, the impact of HIV on the immune system and the role of treatment. In “Body Mapping” (Figure 1), students make life-sized tracings of their bodies as a tool to articulate their understanding of the disease and its social implications. This activity consolidates HIV/AIDS information and also highlights the importance of a strong sense of self and identity. The activity combines scientific and psychosocial approaches.

Teachers are invited to download the students' and teachers' books as well as to share their comments and suggestions for the adaptation of activities in different contexts on our website. Please refer to the Practical guidelines for more detailed information about these resources.

**Box 1. Practical Guidelines**

For a detailed description of how to prepare and implement this activity and others, please refer to the educator's guide, 2006, pp. 29–31, which you can access from http://www.empathytrust.org.

**A Note about Phenolphthalein**

While there may be alternatives, we have found that phenolphthalein is the best option as it only indicates alkaline solutions, whereas litmus paper always shows a colour. Teachers do not need a large quantity of phenolphthalein, and it is easy to give each school a bottle of it at the end of each workshop. It must be remembered that most urban and rural South African schools do not have functional science laboratories.
